# Alexithymia of nursing students in higher vocational college: application of latent profile analysis and network analysis

**DOI:** 10.3389/fpsyt.2026.1854583

**Published:** 2026-06-05

**Authors:** Jianhua Zhang, Ge Liu, Yaxin Zeng, Manxin Zhang

**Affiliations:** 1 School of Physical Education and Arts, Hunan University of Medicine, Huaihua, Hunan, China; 2 Department of Nursing, Heze Medical College, Heze, Shandong, China; 3 Breast Oncology Department, Affiliated Cancer Hospital and Institute of Guangzhou Medical University, Guangzhou, Guangdong, China; 4 Department of Pulmonary and Critical Care Medicine, Nanjing Gaochun People’s Hospital, Nanjing, Jiangsu, China

**Keywords:** alexithymia, internet addiction, mental health, nursing education, physical activity

## Abstract

**Background:**

This study aims to identify different subgroups of alexithymia among vocational college nursing students, explore the critical values and core dimensions of these subgroups, and investigate the potential mechanisms influencing their experiences.

**Method:**

By using the method of convenient sampling, a cross-sectional online survey was conducted among 1,103 vocational college nursing students from 10 universities across the country. Participants completed electronic questionnaires assessing demographics, Toronto Alexithymia Scale (TAS-26), Physical Activity Rating Scale-3 (PARS-3) and Chen Internet Addiction Scale – Revised (CIAS-R). Data analysis was conducted using latent profile analysis (LPA), multiple logistic regression, and network analysis.

**Result:**

Latent Profile Analysis (LPA) revealed three distinct alexithymia profiles among vocational nursing students: low (11.062%), moderate (72.801%), and high (16.138%). Multinomial logistic regression identified female gender and internet addiction as significant factors associated with a higher risk of alexithymia. Furthermore, network analysis delineated the central symptoms for each subgroup: Difficulty identifying feelings emerged as the core symptom in the moderate profile, whereas Lack of fantasy was identified as the central symptom in the high alexithymia profile.

**Conclusion:**

Alexithymia among vocational nursing students is a heterogeneous construct comprising three distinct subtypes. Our findings clarify the key risk factors associated with these subtypes and identify central symptoms that characterize each subgroup. Importantly, given that internet addiction emerged as a significant modifiable risk factor, we recommend that nursing educators and mental health professionals prioritize interventions targeting problematic internet use. Such focused strategies are likely to indirectly enhance students’ emotional awareness and expression, thereby alleviating alexithymic traits across subtypes. These results provide an empirical foundation for developing precise, subgroup-specific psychological support programs.

## 1 Introduction

Alexithymia, also referred to as “emotional expression inability,” is a personality trait first identified in patients with clinical psychosomatic diseases (1). It is characterized by difficulties in identifying emotions, describing and communicating feelings to others, and distinguishing between emotional states and somatic sensations, coupled with a lack of emotion-related fantasy and an externally oriented thinking style (1). The prevalence of alexithymia in the general population ranges between 10% and 18% across different countries (2). Due to impairments in emotional recognition and expression, individuals with alexithymia are more susceptible to various physical and mental disorders (3). Alexithymia and internet addiction share a closely intertwined bidirectional relationship (4). On one hand, individuals with alexithymia, due to difficulties in identifying and expressing emotional and somatic states, are more likely to turn to the internet as a primary source of social support and emotional comfort (4). According to the Uses and Gratifications Theory, online interactions fulfill psychological needs by providing a safer and more comfortable communicative environment, thereby alleviating distress in face-to-face social settings; however, this compensatory reliance may escalate into excessive use and ultimately internet addiction (5). On the other hand, internet addiction can impair real-world emotional cognition, blur self-awareness, and exacerbate interpersonal difficulties, which may further intensify alexithymic traits (6, 7). The Cognitive-Behavioral Model of pathological internet use posits that emotional and cognitive dysregulation itself serves as a key predisposing factor for internet addiction (8). Furthermore, the association between physical activity and alexithymia may operate through direct or indirect pathways. Grounded in the Stimulus-Organism-Response (S-O-R) framework, physical exercise serves as an external stimulus (S) that modulates internal cognitive and physiological states (O), thereby ameliorating difficulties in emotional identification and expression characteristic of alexithymia (R) (9). Previous empirical studies further support this mechanism, indicating that regular physical activity can effectively reduce alexithymia levels (10, 11).

Some studies have found that people with narrative disorders are at risk of having difficulty coping with stress, indicating that emotional disorders are potential risk factors and predictors of certain psychosomatic or mental illnesses (12), especially among nursing students (13, 14). People with alexithymia are prone to exhaustion due to insufficient coping strategies (15), weak social support networks, and an inability to manage their own or others’ emotions (16). Excellent clinical nursing skills and strong humanistic care competencies are key entry points for patients to experience high-quality nursing care (17).

Higher vocational college nursing students, who account for about half of all nursing students in China, are in post-secondary schools that grant non-bachelor’s degrees and offer 2- or 3-year courses, which plays a vital role in the future nursing workforce (18). However, the presence of alexithymia represents a potential psychological factor that impacts nursing students’ physical and mental health, and has been identified as a direct predictor of both somatic and mental disorders (13). Therefore, it is of significant importance to reduce alexithymia, mitigate adverse psychological states, and improve the overall mental health of nursing students.

Alexithymia is a multidimensional construct. Previous studies have often relied on total scale scores to assess alexithymia among nursing students (15). However, students with identical total scores may exhibit distinct patterns of alexithymia, reflected in varied response profiles across different items. Traditional statistical approaches—such as analyzing mean total scores—are often overly simplistic and generalized, failing to capture individual heterogeneity and potentially leading to suboptimal intervention outcomes. Identifying heterogeneous subgroups and their specific characteristics is essential for enhancing intervention effectiveness. Emerging evidence suggests that interventions tailored to groups with similar psychological profiles yield superior results (19, 20). By classifying nursing students into subgroups based on shared alexithymia traits, it becomes possible to design personalized strategies adapted to each subgroup’s unique needs. Latent Profile Analysis (LPA) is a person-centered methodological approach that classifies individuals into distinct subgroups using continuous observed variables, thereby capturing heterogeneity within populations more effectively (21). As a robust method for detecting latent subgroups, LPA can help uncover varying manifestations of alexithymia, enabling nursing educators to develop more targeted and effective interventions. Therefore, applying LPA to identify potential alexithymia subtypes is both necessary and justified.

While prior studies have explored the varied manifestations of alexithymia among nursing students, its core components are distinct yet interrelated (13, 22). To date, however, no research has systematically examined the specific interrelationships among these internal dimensions. Network analysis (NA) provides a framework for visualizing systems as interconnected nodes (variables) and edges (their relationships). To identify central nodes, three key metrics are typically employed: strength, closeness, and betweenness centrality (23). Node strength is the sum of its connection weights. Closeness is the inverse of the average distance to all other nodes, denoting informational efficiency. Betweenness counts how often a node acts as a bridge along the shortest paths between other node pairs (24). This approach allows for a nuanced understanding of system dynamics, such as symptom interactions, and pinpoints optimal targets for intervention (25). Therefore, having identified distinct alexithymia subtypes among nursing students, a deeper profiling of their interrelationships and core characteristics will be instrumental in guiding the design of more effective and targeted psychological interventions.

## 2 Materials and methods

### 2.1 Participants

Between April and May 2025, nursing students were recruited from 10 universities in central-eastern China (Shandong, Hubei, Hunan, and Henan provinces) using a convenience sampling method. Data were collected via anonymous self-administered questionnaires distributed by class instructors. To minimize duplicate submissions, multiple technical and procedural safeguards were implemented: each survey link was bound to a unique participant ID generated by the platform, IP address monitoring was enabled to block multiple submissions from the same source, and participants were explicitly instructed to complete the questionnaire only once. During data cleaning, responses with identical response patterns or completion times shorter than 5 minutes were excluded. To enhance the validity of self-reported data and mitigate response bias, attention-check items were embedded in the questionnaire to detect careless responding. Data collection continued until no new submissions were recorded for seven consecutive days, at which point it was formally terminated. We calculated the sample size as per the following formula:


n=μα22π(1−π)δ2

(where α = 0.05, μα/2 = 1.96, δ = 0.05) (2, 26). In order to ensure sufficient sample size, π = 24.6% was set for calculation (2). Considering 20% non-response rates, we found the minimum sample size was 357. A total of 1,111 questionnaires were received. After excluding 8 responses due to missing data or highly uniform answer patterns, 1,103 valid responses were retained for final analysis.

#### 2.1.1 Inclusion criteria

(1) being a daily higher vocational college nursing student; (2) Mandarin language proficiency sufficient for unhindered communication; (3) Willingness to provide informed consent.

#### 2.1.2 Exclusion criteria

(1) A documented diagnosis of mental disorders (e.g., depression, anxiety disorder, schizophrenia) by a qualified mental health professional, as self-reported via a brief pre-survey health screening questionnaire; (2) Current chronic physical or medical conditions that may affect emotional experience or daily functioning (e.g., chronic pain, neurological conditions, endocrine disorders), also assessed through the same screening questionnaire; (3) Medical leave or academic suspension during the study period.

### 2.2 Measures

#### 2.2.1 Social demographic information

Social demographic information includes gender (male or female), age, living status (urban or rural), monthly household income, Personality [introverted (tending to regain energy through solitude), extroverted (gaining energy through social interaction), and intermediate type (exhibiting balanced traits of both introversion and extroversion, with high situational adaptability)].

#### 2.2.2 Toronto alexithymia scale

Toylor et al. compiled the TAS-26 in 1984 (27). Subsequently, in 1991, Chinese scholar Yao et al. carried out the Chinese translation (28). The scale contains 26 questions and four dimensions: the ability to describe emotions, recognize and distinguish between emotions and body feelings, fantasy, and extroverted thinking. A 5-point scale is used, from 1 (strongly disagree) to 5 (strongly agree), with a total score ranging from 26 to 130. The higher the score, the more severe the alexithymia. The Cronbach’s α coefficients for difficulty describing feelings, difficulty identifying feelings, lack of fantasy, and externally-oriented thinking were 0.761, 0.891, 0.877, and 0.787, respectively. McDonald’s ω values for difficulty describing feelings through externally-oriented thinking were 0.758, 0.883, 0.872, and 0.887, respectively. In the present study, the Cronbach’s α and McDonald’s ω for the overall TAS-26 score were 0.962 and 0.961, respectively.

#### 2.2.3 Physical activity rating scale

Physical exercise was assessed using the Physical Activity Rating Scale-3 (PARS-3), originally revised by Liang (1994) (29) for use in Chinese populations. This instrument assesses physical activity across three domains: intensity, frequency, and duration, each rated on a 5-point scale. Intensity and frequency scores range from 1 to 5 points, while duration is scored from 0 to 4 points. A composite physical activity score (range: 0–100) is derived by multiplying the scores of the three dimensions. Higher total scores indicate more vigorous physical activity levels. The PARS-3 scale has been widely applied in health behavior and epidemiological research (30, 31). In our study, the Cronbach’s α and McDonald’s ω coefficients for the PARS-3 were 0.609 and 0.621, respectively.

#### 2.2.4 Chen internet addiction scale – revised

The Internet Addiction Scale used in this study was originally developed by Chen (32) et al. and later revised for the local context by Bai (33) et al. This self-report instrument comprises 19 items, each rated on a 4-point Likert scale (from 1 to 4). It assesses four dimensions: Compulsive Internet Use and Withdrawal Symptoms (Sym-C & Sym-T), Tolerance (Sym-T), Interpersonal and Health Problems (RP-IH), and Time Management Problems (RP-TM). The scale exhibited excellent internal consistency in the present sample (Cronbach’s *α* = 0.972; McDonald’s *ω* = 0.972).

### 2.3 Statistical analysis

Data analysis was conducted using Mplus 8.3, SPSS 26.0 (IBM, Armonk, NY, USA), and the R statistical software (version 4.3.1). Descriptive statistics were computed for all variables. Continuous variables are described as mean and standard deviation (SD), while categorical variables are summarized using frequencies and percentages.

Latent profile analysis (LPA) was performed to identify distinct subgroups of alexithymia based on item-level responses to the Toronto Alexithymia Scale. Model fit was assessed using the Akaike Information Criterion (AIC), Bayesian Information Criterion (BIC), and sample-size adjusted BIC (aBIC), with lower values indicating superior fit. Classification accuracy was evaluated by entropy, where values closer to 1 are preferred (34). The optimal number of classes was determined by comparing the k-class model against the k-1 class model using the Bootstrapped Likelihood Ratio Test (BLRT) and the Lo-Mendell-Rubin Likelihood Ratio Test (LMRT); a significant p-value for these tests suggests that the k-class model provides a better fit (35).

The chi-square test was used to compare categorical variables across subgroups. For continuous variables that did not meet the assumption of normality, the Mann-Whitney U test was applied to assess differences between subgroups. *Post-hoc* comparisons were conducted using the Bonferroni correction for inter-group analyses. Variables that demonstrated statistically significant differences in the univariate analyses were subsequently entered into a binary logistic regression model.

We constructed a symptom network using polychoric correlations and regularized it with GLASSO combined with the EBIC criterion. This Gaussian Graphical Model (GGM) represents symptoms as nodes and their regularized partial correlations as edges. Central symptoms with high Expected Influence (EI) can activate others in the network (36). We identified bridge symptoms connecting different clusters by calculating Bridge Expected Influence (BEI), where positive values indicate activating and negative values indicate deactivating influences across clusters (36, 37).We ensured the robustness of our findings through bootstrap methods implemented in the R bootnet package. The accuracy of edge weights was confirmed via non-parametric bootstrap (1,500 samples) to calculate 95% CIs (23). The stability of BEI centrality was tested using a case-dropping bootstrap (1,500 samples), yielding a correlation stability (CS) coefficient; values >0.5 are optimal and should not be<0.25 (23). Significant differences in edges and BEI values were determined using bootstrap difference tests (1,500 samples) (23).

## 3 Results

### 3.1 Common method deviation test

Prior to formal analysis, we examined the distributional properties of the total scores for the TAS-26, CIAS-R, and PARS-3. Given the large sample size (*N* = 1,103), the Shapiro-Wilk test was employed as the primary criterion for univariate normality. Results indicated that all three scales met the assumption of normal distribution (*p* = 0.846, 0.934, and 0.788, respectively; all *p* > 0.05). Multivariate normality was subsequently assessed using Mardia’s test. Results indicated that the data met the assumption of multivariate normality in both skewness and kurtosis. Accordingly, maximum likelihood (ML) estimation was employed for parameter estimation. Additionally, to ensure data quality, a data screening strategy was implemented: univariate outliers were identified, and cases with outlier proportions exceeding 5% across key variables were excluded from subsequent analyses to minimize their potential impact. Additionally, to evaluate the potential influence of common method bias (CMB), we conducted Harman’s single-factor test. Exploratory factor analysis extracted five factors with eigenvalues greater than 1. Under the unrotated principal component framework, the first factor accounted for 38.321% of the total variance, which falls below the recommended 40% threshold (38). This result suggests that common method bias is unlikely to substantially confound the interpretation of our findings.

### 3.2 Participant characteristics

A total of 1,103 students from vocational colleges were recruited for this study. The majority of participants were female (81.6%). Over half of the students (57.9%) reported a rural background, and most (84.0%) were not the only child in their family. Detailed characteristics of the participants are presented in **Table 1**.

**Table 1:** Demographic and academic-related characteristics by latent profiles (N = 1103).

Variable	N (100%)	Lower alexithymia traits (122)	Moderate alexithymia traits (803)	Elevated alexithymia traits (178)	χ²/Z	*P*
Gender					11.394	0.003
Male	203	36	135	32		
Female	900	86	668	146		
Age					3.072	0.215
18–19 Year	466	47	352	67		
>20Year	637	75	451	111		
Only Child (OC)					0.392	0.822
Yes	176	21	129	26		
No	927	101	674	152		
Family Residence					4.718	0.095
Urban	464	51	351	62		
Rural	639	71	452	116		
Hold a Position						
Student Union	121	13	90	18	8.654	0.194
Club Officer	52	4	35	13		
Class Committee Member	263	36	178	49		
No	667	69	500	98		
Monthly Household Income(¥)					12.118	0.059
≤3000	228	26	165	37		
3001-6000	492	36	351	90		
6001-9000	224	19	179	26		
≥9001	159	26	108	25		
Alcohol consumption					2.886	0.236
No	1016	109	746	161		
Yes	87	13	57	17		
Smoking					1.023	0.600
No	1070	116	782	172		
Yes	33	6	21	6		
Family Atmosphere					24.266	<0.001
Highly Democratic	452	71	310	71		
Moderately Democratic	552	38	429	85		
Authoritarian	99	13	64	22		
Nursing Interest (NI)					24.764	<0.001
Dislike	241	40	172	29		
Neutral	802	71	599	132		
Like Very Much	60	11	32	17		
Personality					8.105	0.088
Intermediate type	181	28	119	34		
Ambivert	689	74	513	102		
Extroverted	233	20	171	42		
Physical Activity (PA)	_	13.385 ± 16.196	15.168 ± 14.674	15.747 ± 16.983	0.938	0.392
Internet Addiction	_	28.959 ± 11.511	39.473 ± 9.098	43.281 ± 14.098	73.585	<0.001

### 3.3 Latent profile analysis of alexithymia

**Figure 1:**
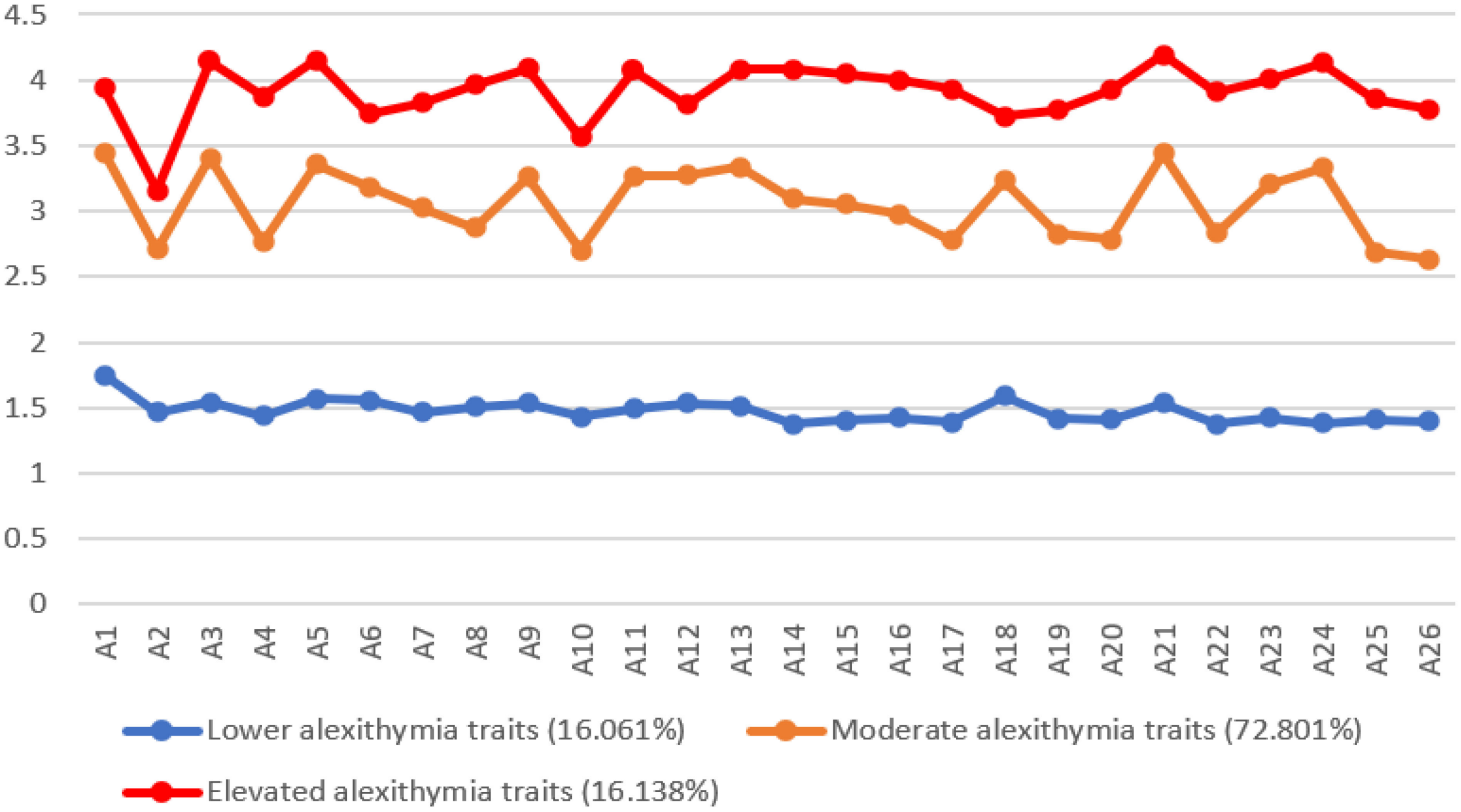
Estimated sample item means for the three latent profiles of childhood trauma.

A latent profile analysis (LPA) was performed using the 26 items of the Toronto Alexithymia Scale (TAS-26) as indicators to identify distinct subgroups of alexithymia among vocational nursing students. Models with one to five latent profiles were estimated and compared (**Table 2**, **Figure 1**). The Akaike Information Criterion (AIC), Bayesian Information Criterion (BIC), and sample-size adjusted BIC (aBIC) decreased consistently as the number of profiles increased from 1 to 5. However, the 5-profile model was rejected because its Lo-Mendell-Rubin adjusted likelihood ratio test (LMR-LRT) was not statistically significant (p > 0.05). The 3-profile solution was selected as the optimal model. This decision was based on a combination of statistical indices and clinical interpretability: although the entropy value for the 3-profile model was above 0.90, indicating excellent classification accuracy, it also provided the most distinct and theoretically meaningful profiles of alexithymia characteristics. The three identified profiles were labeled as: “Lower alexithymia traits” (11.06%), “Moderate alexithymia traits” (72.80%), and “ Elevated alexithymia traits “ (16.14%).

**Table 2:** Model fit indices for latent profile analysis (N = 1103).

Indices	Unconditional model				
	1-profile	2-profile	3-profile	4-profile	5-profile
Fit statistics					
AIC	79127.506	68472.437	64036.825	60624.960	59061.147
BIC	79387.807	68867.894	64567.439	61290.730	59862.073
aBIC	79222.643	68616.971	64230.757	60868.290	59353.875
Entropy	–	0.998	0.978	0.970	0.976
BLRT	–	<0.001	<0.001	<0.001	0.149
LMRT	–	<0.001	<0.001	<0.001	0.151
Group sizes (%)					
C1	100%	11.061%	11.061%	10.789%	5.984%
C2		88.939%	72.801%	57.117%	5.802%
C3			16.138%	16.954%	17.679%
C4				15.141%	55.938%
					14.597%

LL, Log-likelihood. AIC, Akaike Information Criterion. BIC, Bayesian Information Criterion. aBIC, adjusted BIC. LMR, Lo–Mendell–Rubin likelihood ratio test.

### 3.4 Demographic and academic-related characteristics of each profile

The results of the single-factor analysis showed that gender, family Atmosphere, nursing Interest. There was a statistically significant difference in Internet Addiction (P< 0.05).

### 3.5 Predictor of latent profile membership

Using the “Lower alexithymia traits” group as the reference category, multinomial logistic regression was performed to identify predictive factors (**Table 3**). The results indicated that being male (OR = 0.485) was associated with a significantly higher likelihood of belonging to the “Lower alexithymia traits” group than to the “Moderate alexithymia traits” group. Furthermore, being male (OR = 0.458) and the absence of Internet Addiction (OR = 0.146) were significant predictors for membership in the “Lower alexithymia traits” group relative to the “High Alexithymia” group.

**Table 3:** Multiple logistic regression analysis of influencing factors of alexithymia among undergraduate nursing students (N = 1103).

Variables	Moderate alexithymia traits vs lower alexithymia traits				Elevated alexithymia traits vs lower alexithymia traits			
	β	SE	OR	*P*	β	SE	OR	*P*
Gender (reference: Female)								
Male	-0.724	0.227	0.485	0.001	-0.781	0.291	0.458	0.007
NI (reference: Like Very Much)								
Dislike	-0.594	0.435	0.552	0.142	0.461	0.478	1.586	0.334
Neutral	0.355	0.253	1.426	0.125	0.642	0.304	1.901	0.035
Family Atmosphere (reference: Authoritarian)								
Highly Democratic	0.073	0.343	1.075	0.832	-0.044	0.411	0.957	0.914
Moderately Democratic	0.786	0.355	2.194	0.027	0.418	0.417	1.519	0.316
Internet Addiction (reference: Yes)								
No	-0.806	0.484	0.446	0.096	-1.926	0.502	0.146	<0.001

### 3.6 Network analysis across latent profiles

**Figure 2:**
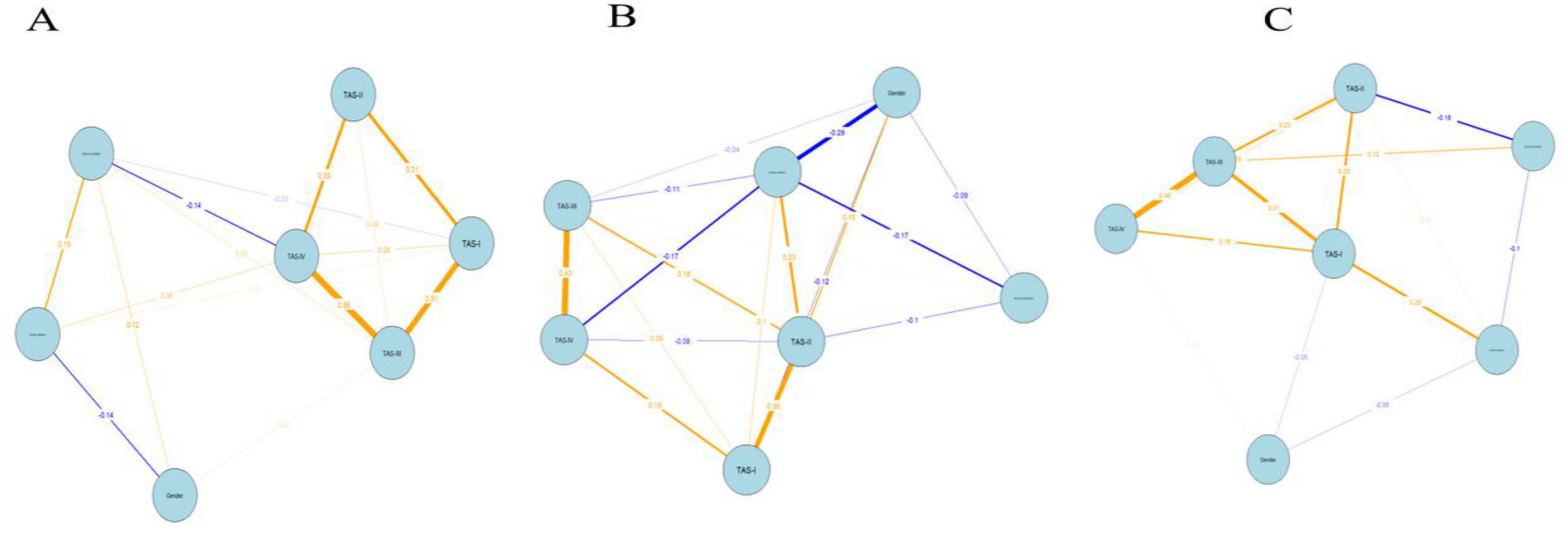
The networks of the three latent profiles. **(A)** lower alexithymia traits group **(B)** moderate alexithymia traits group; **(C)** elevated alexithymia traits group. TAS-I: difficulty describing feelings; TAS-II: difficulty identifying feelings; TAS-III: lack of fantasy; TAS-VI: externally-oriented thinking. Note: yellow indicates a positive correlation, while blue indicates a negative correlation. The thickness of the edge reflects the magnitude of the correlation.

**Figure 3:**
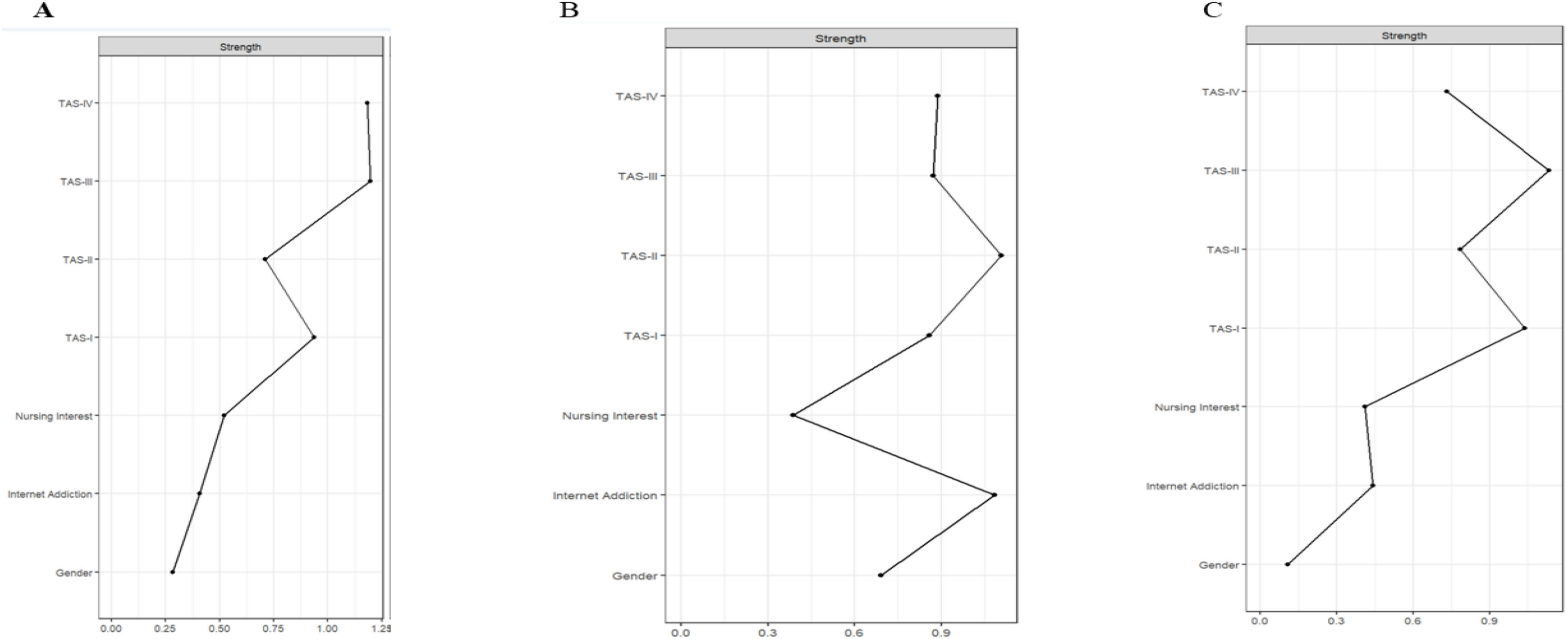
The strength centrality index of the networks of the three latent profiles. **(A)** lower alexithymia traits group **(B)** moderate alexithymia traits group; **(C)** elevated alexithymia traits group. TAS-I: difficulty describing feelings; TAS-II: difficulty identifying feelings; TAS-III: lack of fantasy; TAS-VI: externally-oriented thinking.

The network structure and centrality indices for the four alexithymia dimensions and their influencing factors are depicted in **Figures 2**, **3**, respectively. **Figure 2A** illustrates the network for the “Lower alexithymia traits” subgroup. The analysis revealed robust connections between Lack of Fantasy and Externally-Oriented Thinking (edge weight = 0.56), as well as between Lack of Fantasy and Difficulty Describing Feelings (edge weight = 0.51). Regarding influencing factors, interest in the nursing major was negatively associated with Externally-Oriented Thinking (r = -0.14). Furthermore, as shown in **Figure 3**, the strength centrality analysis identified Lack of Fantasy as the most central node in this subgroup (strength = 1.1965).

**Figure 2B** presents the symptom network for the “Moderate alexithymia traits” subgroup. The network analysis revealed two notable symptom associations: a positive connection between Externally-Oriented Thinking and Lack of Fantasy edge weight = 0.43), and a link between Difficulty Describing Feelings and Difficulty Identifying Feelings (edge weight = 0.36). Regarding influencing factors, Internet Addiction demonstrated a negative correlation with Externally-Oriented Thinking (r = -0.17). Furthermore, as detailed in the centrality plot (**Figure 3**), Difficulty Identifying Feelings was identified as the most central node within this subgroup’s network, exhibiting the highest strength centrality value of 1.1074.

**Figure 2C** illustrates the network structure for the “Elevated alexithymia traits” subgroup. Network analysis revealed significant positive correlations between Externally-Oriented Thinking and Lack of Fantasy (edge weight = 0.46), as well as between Difficulty Describing Feelings and Lack of Fantasy (edge weight = 0.31). Regarding influencing factors, Internet Addiction showed a positive association with Difficulty Describing Feelings (r = 0.25). Furthermore, strength centrality analysis identified Lack of Fantasy as the core dimension in this subgroup (strength = 1.132), as shown in **Figure 3**.

## 4 Discussion

### 4.1 Subgroups of alexithymia among vocational nursing students

Building on recent person-centered approaches in nursing research, this study utilizes LPA to examine the heterogeneity of alexithymia specifically within a sample of vocational nursing students. Using this person-centered approach, three distinct subgroups were identified: a “Lower alexithymia traits” group (11.061%), a “Moderate alexithymia traits” group (72.801%), and a “Elevated alexithymia traits” group (16.138%).

The most pronounced increasing trends in the high and Moderate alexithymia traits subgroups were observed for items 1, 3, 5, 9, 13, 21, and 24. Results indicated that the TAS-VI dimension (externally oriented thinking) scored the highest among all dimensions. This cognitive style is characterized by a primary focus on external events, with minimal attention allocated to internal feelings, emotional states, and fantasies (1). Such an outward-focused attentional pattern contributes to a relatively impoverished inner emotional life and a tendency to engage with the world in a concrete, utilitarian manner, rather than through introspective emotional processing (1). This finding is consistent with previous reports (39). Vocational nursing students are typically in the transitional phase from adolescence to early adulthood, a period during which emotional cognition and metacognitive abilities are still developing. They may not yet have fully acquired the skills necessary to identify, describe, and integrate complex internal feelings (40). Consequently, their cognitive resources may naturally gravitate toward the more familiar and manageable external environment (40). Furthermore, individuals with high levels of alexithymia, due to deficits in emotional perception, often fail to develop effective coping strategies and tend to rely on maladaptive defense mechanisms (41). The use of immature defense mechanisms, in turn, is a recognized risk factor for various psychological issues, including suicidal ideation and eating disorders (41). Therefore, identifying the influencing factors of alexithymia in this population and implementing targeted interventions are of crucial importance.

### 4.2 Factors influencing latent classes of alexithymia in vocational nursing students

Our study identified gender as a significant influencing factor in latent profiles of alexithymia among vocational nursing students. This finding is partially consistent with previous literature (2, 42, 43); however, the specific pattern we observed—a higher prevalence of alexithymia among female nursing students—diverges from some existing reports (44, 45). This discrepancy may be explained by underlying socio-cultural mechanisms. Men are often socialized to internalize psychological distress, which may lead to different manifestations of symptomatology (46). In contrast, women may be more susceptible to the cumulative amplification of emotional and behavioral issues over time, potentially contributing to their higher observed rates of alexithymia (46). Therefore, it is imperative for educators to develop early identification and intervention strategies specifically tailored for female nursing students. The implementation of targeted psychological support programs could effectively mitigate the progression of alexithymia, thereby reducing its negative impact on student well-being. Such proactive measures are essential for cultivating a mentally healthy and resilient future nursing workforce.

Our findings indicate that vocational nursing students with internet addiction are more likely to fall into the moderate or high alexithymia subgroups. This result aligns with previous research conducted among university students (47). Over-dependence on the internet may divert time and attention away from face-to-face communication and emotional exchange in real life, which is associated with elevated alexithymia traits among vocational nursing students (48). Higher levels of internet dependency are associated with greater difficulty in identifying and describing emotions. Furthermore, individuals with pronounced internet addiction tendencies often exhibit specific personality traits that, over time, may impair both emotional expressivity and the ability to differentiate between distinct feelings (49).

### 4.3 NA of alexithymia in the three latent subgroups of vocational nursing students

NA helps identify central nodes within psychological constructs, where nodes with high strength centrality exert a disproportionately strong influence on the entire network. Targeting these core components in psychological interventions is strategic, as changes in them can propagate to peripheral associated symptoms, potentially reducing or eliminating the manifestation of co-occurring issues (50). Our centrality analysis of the alexithymia construct revealed both common and distinct central features across the three latent subgroups. In the Moderate alexithymia traits network, Difficulty identifying feelings emerged as the pivotal node, suggesting it may represent a core characteristic of this subgroup. Furthermore, lack of fantasy ranked second in the moderate subgroup and was identified as a central component in the high alexithymia subgroup, indicating its transdiagnostic importance. Interventions targeting these central components are likely to produce cascading effects, initially influencing adjacent nodes and then permeating the broader network. Therefore, developing subgroup-specific interventions that address these distinct central features offers a promising and efficient approach to alleviating alexithymia among vocational nursing students. Our findings provide a novel, data-driven perspective for nursing educators in selecting precise intervention content and formulating targeted strategies.

### 4.4 Implication for practice

This study confirms that vocational nursing students experience alexithymia at varying levels of severity, underscoring the need for tailored interventions. Network analysis identified distinct central symptoms within the three established subgroups, providing precise targets for prevention and treatment. Furthermore, female gender and internet addiction were identified as high-risk factors. Collectively, these findings offer crucial insights for nursing educators, informing the development of targeted strategies to mitigate the prevalence and impact of alexithymia. Specifically, we recommend implementing emotion regulation skills training to enhance students’ capacity to identify, process, and verbalize emotions, thereby fostering greater empathy during direct patient care (51). Furthermore, incorporating interpersonal communication skills training into the nursing curriculum is expected to strengthen the therapeutic alliance between nurses and patients (52, 53). By translating these network-derived insights into practical, skill-based educational programs, nursing institutions can better equip future clinicians to manage their emotional challenges while delivering high-quality, empathetic patient care.

### 4.5 Limitations

This study has several limitations that warrant careful consideration. First, the cross-sectional design precludes causal inference and prevents the establishment of temporal precedence among variables. Although existing literature suggests bidirectional relationships between alexithymia, substance use, and internet addiction, our design cannot determine whether these behaviors precede, follow, or mutually reinforce alexithymic traits. Additionally, potential confounders such as underlying mental or physical health conditions and patterns of substance use/abuse were not controlled for and may have influenced the observed associations. Second, the reliance on self-report measures renders the data susceptible to reporting biases, including social desirability and recall bias. Third, the use of the TAS-26 rather than the TAS-20, which possesses more established psychometric properties, may introduce certain measurement constraints. Finally, while physical activity is generally beneficial for mental health, its limited capacity to differentiate alexithymia subtypes suggests that alexithymia operates primarily as a cognitive-affective trait, with overall activity volume offering modest discriminatory power. Specific dimensions of physical activity—such as type, intensity, duration, or social context—may provide greater explanatory value. To address these limitations, future research should employ longitudinal or experimental designs to clarify temporal dynamics, control for key clinical and behavioral covariates, utilize standardized assessment tools, and cross-validate data-driven profiles with clinically established cut-off criteria. These steps will enhance the interpretability and generalizability of findings across diverse nursing populations.

## 5 Conclusion

In summary, this study identified three distinct alexithymia subgroups among vocational nursing students, underscoring the necessity for precise intervention. Key risk factors included female gender and internet addiction. Furthermore, network analysis revealed divergent central symptoms across these subgroups. Consequently, we emphasize the urgent need for nursing educators to develop and implement tailored strategies that address the unique characteristics and core deficits of each specific subgroup.

## Data Availability

The original contributions presented in the study are included in the article/supplementary material. Further inquiries can be directed to the corresponding author.
